# Senolytics and senostatics as adjuvant tumour therapy

**DOI:** 10.1016/j.ebiom.2019.01.056

**Published:** 2019-02-06

**Authors:** Susan Short, Edward Fielder, Satomi Miwa, Thomas von Zglinicki

**Affiliations:** a Leeds Institute of Cancer and Pathology, Wellcome Trust Brenner Building, St James's University Hospital, Beckett St, Leeds LS9 7TF, UK; b Newcastle University Institute for Ageing, Institute for Cell and Molecular Biology, Campus for Ageing and Vitality, Newcastle University, Newcastle upon Tyne NE4 5PL, UK

**Keywords:** Cancer, Glioma, Senolytics, Senostatics, Therapy, Survivor

## Abstract

Cell senescence is a driver of ageing, frailty, age-associated disease and functional decline. In oncology, tumour cell senescence may contribute to the effect of adjuvant therapies, as it blocks tumour growth. However, this is frequently incomplete, and tumour cells that recover from senescence may gain a more stem-like state with increased proliferative potential. This might be exaggerated by the induction of senescence in the surrounding niche cells. Finally, senescence will spread through bystander effects, possibly overwhelming the capacity of the immune system to ablate senescent cells. This induces a persistent system-wide senescent cell accumulation, which we hypothesize is the cause for the premature frailty, multi-morbidity and increased mortality in cancer survivors.

Senolytics, drugs that selectively kill senescent cells, have been developed recently and have been proposed as second-line adjuvant tumour therapy. Similarly, by blocking accelerated senescence following therapy, senolytics might prevent and potentially even revert premature frailty in cancer survivors.

Adjuvant senostatic interventions, which suppress senescence-associated bystander signalling, might also have therapeutic potential. This becomes pertinent because treatments that are senostatic in vitro (e.g. dietary restriction mimetics) persistently reduce numbers of senescent cells in vivo, i.e. act as net senolytics in immunocompetent hosts.

## 1 Introduction

Improvements in cancer treatment have rendered many common cancers curable in a high proportion of patients. Although cancer remains a common disease, affecting an estimated 18 million of the world population in 2018, cancer-specific mortality has dropped sharply in the last few decades in developed countries. For example >70% of patients with breast cancer can now expect to live >10 years from diagnosis and many haematological and paediatric cancers have high cure rates [1]. Although efforts continue to address how to improve survival in harder to treat cancers, prominently including brain tumours, there is now greater awareness of health issues in long-term survivors, and in some fields the emphasis has started to shift towards efforts to improve the quality of survivorship after successful cancer treatment [2]. The mainstays of adjuvant treatment i.e. radiotherapy and chemotherapy cause not only short-term toxicities in tissues which rely on continued self-renewal such as bone marrow, GI tract, gonads and skin. They also result in long-term morbidity in a much wider range of organ systems, including cardiovascular, gastrointestinal, pulmonary, hepatic, musculoskeletal and neurological effects as well as enhanced frailty and mortality, together resembling accelerated ageing [3,4]. Although more refined approaches to treatment, particularly advanced surgical and radiotherapy techniques can abrogate some local effects, addressing the long-term attrition of tissue and organ homeostasis as a result of treatment is an unmet research need. The role of senescent cell populations in this context is a relatively under-explored area of research. However there is increasing evidence that promotion of senescence in normal tissues is associated with long term reduced function expressed clinically as an ageing-like phenotype.

There are still major tumour types with very low rates of survival. Malignant gliomas, for example, remain amongst the most lethal of cancers. Median survival for patients suffering from its most common type in adults, glioblastoma, is little over a year despite combination treatment of maximal surgery, high dose radiotherapy and chemotherapy. Despite many attempts at improving outcome using novel agents, the treatment of this disease has not improved in more than a decade [[5], [6], [7]]. These primary brain tumours exemplify the relatively unusual situation where there is *always* significant residual disease post surgery. It is also well established that the brain represents an immune privileged site, where immune-mediated removal of microscopic disease is limited, leaving a large number of cells that can only be ablated by chemo-radiotherapy. Mechanisms of treatment resistance are still poorly understood, but a pool of cells with stem like features associated with up-regulated DNA repair mechanisms and a highly migratory phenotype are thought to represent a resistant population that survive and re-populate the tumour after cytotoxic treatments [[8], [9], [10]]. Definition of novel targeting strategies to alter this treatment-resistant phenotype is a major unmet need in neuro-oncology. Based on evidence, discussed below, that senescence may be particularly relevant in promoting frailty after brain radiotherapy and data supporting senescence in glioma cells after both radiation and chemotherapy, we suggest that brain tumours represent an excellent clinical model in which to investigate senescence as a therapeutic target.

Although outcome in the most common type of high grade glioma in adults remains poor, recent molecular pathology analyses show that there is also a very good prognosis sub-group defined by 1p19q chromosomal deletion and IDH mutation [11,12]. This molecular classification selects patients whose tumours are chemo and radiation sensitive, and who have median survivals >10 years after radiotherapy and adjuvant chemotherapy. In the context of these outcomes, long-term toxicity of treatment is a growing concern in these patients, in which follow up demonstrates cognitive decline in >50% of cases. In a large cohort of long-term childhood cancer survivors, frailty and pre-frailty incidence was highest in CNS cancer survivors [13]. Recent data suggest that normal brain tissue, particularly hippocampus, is sensitive to even low doses of radiation when neurocognitive change is used as an end-point, implying that despite advances in highly targeted radiotherapy, novel approaches to ameliorate the effects of radiotherapy on normal brain remain a significant unmet need [14,15].

This review suggests that cell senescence is an essential driver for both tumour relapse following radio- and chemotherapy and for premature ageing in cancer survivors and summarizes the evidence that both can be treated by senolytic as well as senostatic interventions.

## 2 Cell senescence

Cell senescence has originally been identified as the irreversible and reproducible loss of proliferative capacity of human somatic cells in culture [16]. However, a more appropriate definition is that of a cellular stress response [17], characterized by the integration of at least three interacting signalling pathways, namely i) a persistent DNA Damage Response (DDR) [18] frequently initiated by shortened or otherwise uncapped telomeres [19]. The DDR activates ii) senescence-associated mitochondrial dysfunction (SAMD) typically characterized by decreased respiratory activity and membrane potential together with increased mitochondrial ROS production [20,21]. SAMD might be driven or at least enhanced by dysregulated mitophagy in senescence [22,23]. Thirdly, senescent cells are characterized by a senescence-associated secretory phenotype (SASP, see [24] for a recent review). Following induction of senescence, the SASP develops kinetically: In the early phase (coinciding with development of the SAMD) upregulated NOTCH1 signalling causes repression of C/EBPβ and upregulation of an immunosuppressive and pro-fibrotic SASP with high TGF-β levels, followed by later downregulation of NOTCH1 signalling and induction of a C/EBPβ− and NF-κB-driven SASP with high levels of pro-inflammatory interleukins, cytokines and matrix metalloproteases [[25], [26], [27], [28]]. The pro-inflammatory SASP and the SAMD are closely interrelated by positive feedback loops [20,27,28]: Deletion of mitochondria from senescent cells [29] or ROS scavenging [20,30] suppresses the complete senescent phenotype including NF-κB-dependent interleukin production. Conversely, persistent activation of the NF-κB-driven SASP aggravates ROS production and DNA damage in senescent cells [31]. Both SASP and SAMD are further interconnected with a re-wiring of the epigenome [32] and de-sensibilisation of mTOR-dependent nutrient signalling leading to enhanced autophagy activity together with decreased mitophagy [23]. Global epigenetic reprogramming, especially repressive histone H3 lysine 9 trimethylation (H3K9me3) marks in the vicinity of S-phase entry-relevant gene promoters, stably maintains the senescent growth arrest in oncogene- and stress-induced senescence [33]. At the same time, epigenetic reprogramming conveys a more stem cell-like gene expression pattern to senescent cells [[32], [33], [34], [35]].

Importantly, activation of these stress response pathways can often be uncoupled from cell cycle arrest [36]. Firstly, the senescent phenotype develops kinetically over a couple of weeks following an inducing stress event towards ‘deep’ senescence, which is characterized by a stable proliferation arrest in primary cells [20,22,26]. Tumour cells however can escape even from a growth arrest with multiple features of ‘deep’ senescence [36]. Importantly, at least in some models these escaping tumour cells retain epigenetic features developed during senescence, especially increased ‘stemness’ [35]. Conversely, in an ageing organism, postmitotic cells including neurons [37], retinal cells [38], skeletal muscle fibres [39] or cardiomyocytes (unpublished) increasingly display the same sets of markers that characterize cell senescence, indicating upregulation of the same interlinked pathways as in conventional senescence. Senescence markers in post-mitotic cells are activated in response to a persistent DDR [37]. Thus, post-mitotic cells are not inherently senescent, but can become ‘senescence-like’ if they accumulate DNA damage, which might happen many years after they became post-mitotic. Therefore, we propose to use the terminus ‘cell senescence’ to characterize the stress response phenotype irrespective of whether it is coupled with induction of cell cycle arrest or not.

**Fig. 1:**
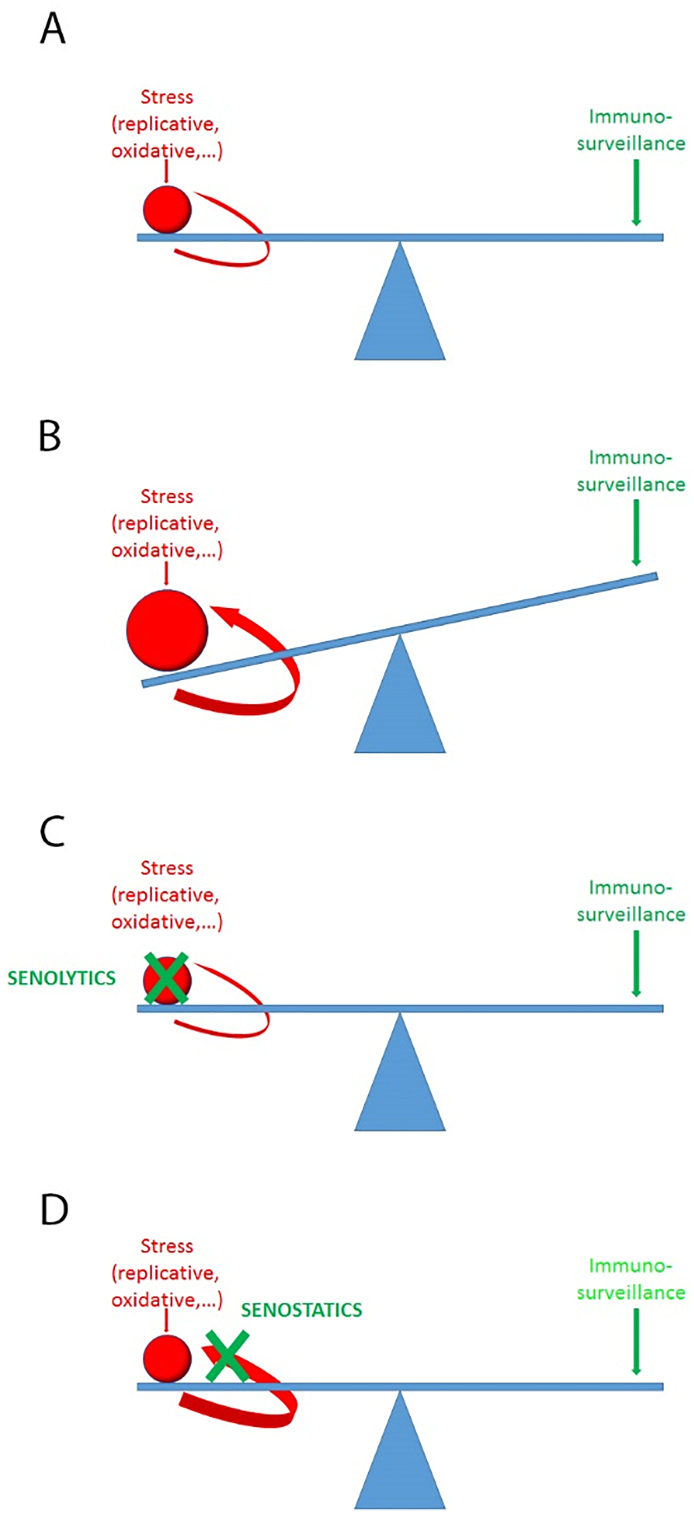
The bystander effect shifts the balance between generation and surveillance of senescent cells. A) In young animals, immune-mediated turnover compensates for cell-autonomous (stress-induced) generation of senescent cells as well as for a minor bystander effect. B) Temporal induction of senescence (e.g. by tumour therapy) or decreasing efficiency of immunosurveillance disturb the balance causing growth of the senescent cell fraction. The bystander feedback will aggravate the imbalance, even if immunosurveillance would not further decline with age. C) By killing a significant fraction of senescent cells, senolytics also reduce bystander signals and regenerate a steady state with low senescent cell numbers. D) Senostatics suppress the bystander effect and enable the immune system to reduce senescent cell frequencies.

Senescence is more than a cell-autonomous stress response. Bioactive molecules released from senescent cells are potent inducers of cell senescence in bystander cells [40,41]. NF-κB-dependent SASP components are sufficient to aggravate autocrine and paracrine senescence [31,42], however, it appears highly probable that additional factors released from senescent cells including exosomal miRNAs [43,44] and pro-oxidants [30,40,45] may also contribute to senescence-induced bystander senescence. Recent data show the relevance of the bystander effect for the accumulation of senescent cells and development of functional defects with age: Injection of small numbers of senescent cells into muscle or skin of mice resulted first in locally enhanced senescence of tissue-resident cells, which was not seen when non-senescent cells were transplanted [39]. Transplantation of slightly larger numbers of senescent cells into visceral fat (still not more than about 0.1‰ of all fat cells in the recipients) was sufficient to cause increased senescence organism-wide, resulting in persistent physical dysfunction [46]. Importantly, these consequences of senescent cell transplantation became evident at a time point when practically all transplanted senescent cells had already been cleared from the recipient tissues [39,46], indicating that senescence-induced bystander senescence is a significant cause for accumulation of senescent cells in vivo. This was confirmed comparing accumulation rates of native senescent hepatocytes in ‘normal’ and multiple immunocompromised mice during ageing and under dietary restriction [39]. These data suggest that a ‘one-off’ localised induction of senescence (e.g. by chemo- or irradiation tumour therapy) may be expected to result in a continuously accelerated accumulation of senescent cells. Conversely, ablation of senescent cells should not just reduce senescent cell numbers but in addition reduce rates of senescent cell accumulation to a youthful state (Fig. 1).

## 3 Senolytics versus senostatics

In addition to the activation of stress responses, senescent cells are characterized by the induction of multiple anti-apoptotic pathways. In recent years, an increasing number of drugs has been identified that inhibit those pathways, inducing apoptosis more or less specifically in senescent cells [[47], [48], [49], [50]]. So far, these senolytic drugs (or drug combinations) show significant variation in their cell type specificity due to differential use of anti-apoptotic mechanisms in senescent cells originating from different cell types. However, a good number of publications report a significant overlap in the beneficial effects of first-generation senolytics amongst each other and with pharmacogenetic approaches that specifically ablate p16-overexpressing cells (Table 1). While there are first data indicating that a single dose of senolytic can result in long-lasting physical improvement, these data were generated in mice transplanted with senescent cells [46], and strong evidence for a curative effect of a single course of senolytic intervention is as yet missing.

**Table 1:** Ageing phenotypes that have been improved by anti-senescence intervention. Pharmacogenetic approaches are underlined, pharmacologic treatments are shown in *italics*.

Examined conditions	Treatment	Reference
Premature muscle weakness, cataract, lipodystrophy	INK-ATTAC	[51]
Cardiovascular function, radiation-induced muscle weakness	*D+Q*	[49]
Lipodystrophy	INK-ATTAC	[52]
Median lifespan, Tumour incidence, Cardiac stress tolerance	INK-ATTAC	[53]
Atherosclerosis	3-MR, INK-ATTAC, INK-NTR, *ABT263*INK-ATTAC, *D+Q*	[54]
		[55]
Irradiation-induced haematoxicity, age-related HSC dysfunction	*ABT263*	[56]
Pulmonary fibrosis	ARF-DTR	[57]
	INK-ATTAC, *D+Q*	[58]
Lung emphysema	*ABT263*	[59]
	ARF-DTR	[60]
Liver steatosis (incl. Human correlative data)	INK-ATTAC, *D+Q*	[61]
Osteoarthritis (incl. Human ex-vivo intervention)	3-MR, *ABT263*	[62]
osteoporosis	*ABT263*	[63]
Chemotherapy-induced multimorbidity (incl. human correlative data)	3-MR	[64]
Chemotherapy-induced liver toxicity Age-related frailty, renal function loss	*FOXO4-DRI*	[65]
Age-related pathology, lifespan	*fisetin*	[50]
Tau-dependent neurodegeneration	INK-ATTAC, *ABT263*	[66]
Obesity-induced anxiety	INK-ATTAC, *D* *+* *Q*	[67]

In contrast to senolytics, senostatics do not kill senescent cells but inhibit paracrine signalling and thus block the ‘proliferation’ of senescence due to the bystander effect. Antioxidants or inhibitors of NF-κB can be efficient senostatics [30,40], and there is evidence that multiple flavonoids, polyphenols and other phytochemicals may have senostatic activity [68,69]. Given the essential role of SAMD for the development of the senescent phenotype including the SASP [29,30], mTOR pathway inhibitors and other interventions that improve mitochondrial (especially complex I) function have significant senostatic potential. This includes rapamycin and other mTOR inhibitors [70], metformin [[71], [72], [73]] and prominently dietary restriction [74]. It is interesting to note that while none of these interventions ablates senescent cells in in- vitro assays, short-term (2 to 4 months) treatment of mice with either rapamycin [75], metformin (Miwa et al., in prep) or dietary restriction [39,61,74] decreased frequencies of cells positive for multiple senescence markers below the levels measured before the intervention. This was dependent on intact immunosurveillance: in contrast to wild-type mice, dietary restriction of severely immunocompromised NSD mice resulted only in a slowing of accumulation of senescent hepatocytes in the liver, but not an actual decrease of their numbers [39]. Importantly, reduction of senescent cell frequencies under dietary restriction remained irreversible (at least for three months) when animals were returned to ad libitum feeding [61]. This suggested that senostatic drugs might actually exert a net senolytic effect in immunocompetent hosts (Fig. 1). If this is true, one might expect similar beneficial effects of relatively short senostatic interventions as with senolytics. This would be advantageous given the on average better safety profiles of senostatic drugs. However, essential experiments are yet lacking; the persistence of reduced frequencies of senescent cells after short or medium term senostatic interventions other than dietary restriction has not been shown. However, there is good evidence for long-term beneficial healthspan effects following not only short-term dietary restriction [76,77] but also short-term rapamycin treatment [78].

## 4 Senescence as tumour suppressor and tumour promoter

In a tumour context, senescence plays important roles. Overexpression of oncogenes in otherwise normal cells triggers oncogene-induced senescence via replicative stress [79,80]. This constitutes an important natural tumour suppression mechanism [81,82] (see [83] for a recent review). Furthermore, DNA-damaging treatments induce not only apoptosis but also senescence in tumour cells [84]. This therapy-induced senescence was shown to contribute significantly to successful chemotherapy outcomes in mouse tumour models [85], not only by inhibiting tumour cell proliferation but also by triggering an immune response eliminating neoplastic (senescent and non-senescent) cells [86]. Accordingly, senescence-inducing drugs (specifically CDK4/6 inhibitors) have shown promise in mouse xenografts [87] and in clinical anti-cancer studies [88] and novel drugs that trigger senescence are being sought [89]. However, one or more of the major cell cycle check point pathways (p53-, p21-, p16-, Cdc2/cdk1- or pRb-dependent) are usually genetically or epigenetically downregulated in cancer cells. While this generally reduces efficiencies of therapy-induced senescence and apoptosis, multiple pathways still remain active for senescence induction in check point-compromised tumour cells, including for instance metabolic [90] or cytosolic DNA sensing [91]. However, suppression of typical cell cycle check points will compromise the stability of the senescent growth arrest. Accordingly, tumour cells can escape from therapy-induced senescence with relative ease [36,[92], [93], [94]]. Unfortunately, these ‘escapers’ may have gained increased tumorigenic and possibly metastatic potential [33,35,36] by both cell-autonomous and non-autonomous mechanisms.

Cell-autonomously, it has been well established that senescence drives epigenetic and gene expression changes that overlap to a significant amount with those found in cancer [95]. Thus, epigenetic reprogramming during induction of senescence might promote more aggressive growth in cells that manage to escape from proliferation arrest. In fact, when senescence was induced in B-cell lymphoma from Eμ-MYC transgenic mice, Wnt signalling pathway and stem cell markers were upregulated. If these cells were released from senescence, they showed strongly enhanced, Wnt-dependent clonogenic growth potential and a much higher tumour initiation potential in vivo. Temporary enforcement of senescence reprogrammed non-stem leukaemia cells into self-renewing, leukaemia-initiating stem cells in a mouse model [35].

In addition, DNA-damaging and senescence-inducing therapies also induce senescence in somatic cells surrounding the tumour, both via direct hits, especially in systemic therapies, and via bystander effects [96]. While the senescent proliferation arrest in stromal bystander cells is stable, senescence is spread around as a bystander effect, causing accelerated ageing in mice over the long term [39,46]. Importantly, cancer cells respond differently to bystander signals: Instead of senescence and growth arrest, SASP signals from senescent tumour or niche cells cause epithelial-to-mesenchymal transition and enhanced proliferation of (pre-)cancerous cells [97,98]. The pro-inflammatory cytokines IL-6 and IL-8 have been shown to control liver tumour progression in mice [99]. The early SASP component TGFβ is specifically efficient as inducer of EMT [100,101]. Moreover, cancer cell invasiveness is supported by the secretion of multiple matrix metalloproteinases as part of the SASP [102], which facilitate translocation of cancer cells from their original place. Thus, despite their major therapeutic benefits, DNA-damaging tumour therapies do create niches that are more supportive for tumour (stem) cell growth [103]. Moreover, the system-wide spread of bystander senescence enables the creation of more effective niches away from the original tumour even after localised therapy.

So far, the relative importance of cell-autonomous (tumour cell) senescence versus non cell-autonomous (niche) senescence for tumour relapse has just begun to be addressed in mouse models. Their importance for tumour therapy in humans is still wide open.

## 5 Anti-senescence as adjuvant tumour therapy

**Fig. 2:**
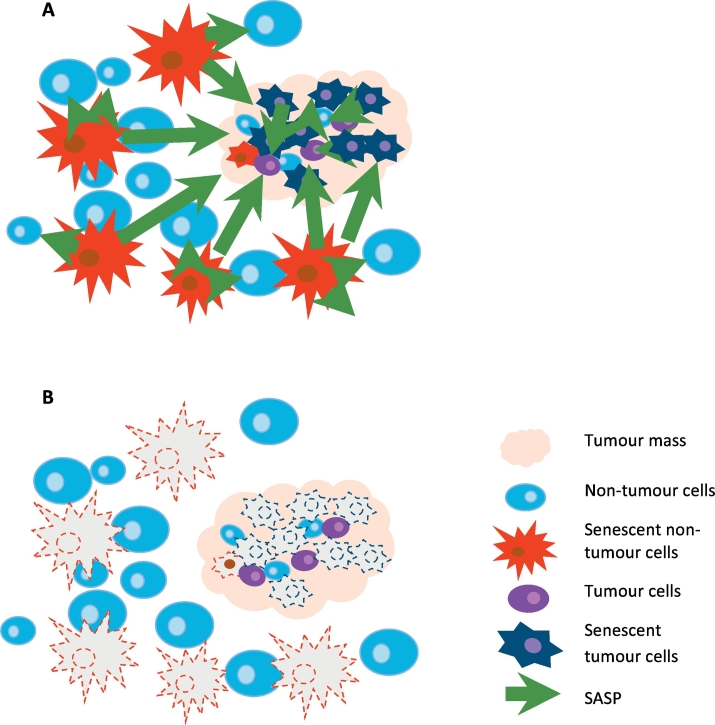
Senolytics complement adjuvant tumour therapy. A) Following irradiation and/or chemotherapy, senescent tumour and non-tumour (niche) cells are generated that secrete SASP factors which promote tumour growth and perpetuate senescence. B) Post-treatment with senolytics removes these sources of tumour regrowth and relapse.

As summarized in Section 4 above, senescence induction is a two-edged sword in tumour therapy: While it frequently, and often significantly, contributes to therapeutic inhibition of tumour growth, it also prepares the ground for relapse with potentially enhanced tumourigenicity. We propose that this may present a specifically serious problem in tumours where anatomical constraints prohibit surgical removal, making radio- and chemotherapy first and main therapeutic interventions, for instance high-grade invasive glioblastoma. Recognizing the problematic of tumour and niche cell senescence in therapy, recent reviews suggested using senolytics as secondary therapy to selectively kill the senescent cells generated by the first line (adjuvant) DNA-damaging therapies [83,89,103,104] (Fig. 2). The potential advantages are impressive: present best-use therapies can be used unmodified and would just need to be followed by a senolytic intervention, which, targeted to long-term non-dividing cells, could be done as a one-off treatment and would not be expected to suffer from adaptation and drug resistance.

Unfortunately, at present the actual experimental and clinical evidence that such an approach can make a difference is yet very slim. Wang [89] identified a number of novel compounds to induce senescence in tumour cells, and showed that one of the known senolytics, ABT263, which targets BCL-2 family-dependent anti-apoptotic pathways, can kill a range of senescent cancer cells, independent of how senescence was induced. An in vivo study showed that elimination of chemotherapy-induced senescent cells by ABT263 reduced cancer recurrence and metastasis in mouse models [64]. This study also showed that chemotherapy induced senescence specifically in non-tumour cells promoted growth and metastasis of implanted tumour cells, and that the senolytic drug ABT 263 was able to reduce them. However, in another study [105] the senolytic cocktail dasatinib + quercetin did not induce apoptosis in senescent hepatocellular carcinoma (HCC) cells and did not reduce the growth of doxorubicin-treated xenotransplanted HCC beyond the effect of doxorubicin alone.

Many senolytics induce apoptosis not only in senescent cells but also (albeit with reduced efficiency) in non-senescent cancer cells. One example is navitoclax (ABT 263), which causes considerable toxicity including thrombocytopenia [106] and has been used with limited success and significant side effects in clinical trials in patients with leukaemia and lymphoma (NCT00406809), lung (NCT00445198) and other cancers. Interestingly, its ability to enhance the activity of other chemotherapeutic agents, even in tumours where navitoclax had no single agent activity, was recognised already at its first description [106]. However, it is still not clear whether this is due to its senolytic activity. In general, results from clinical studies in which senolytics have been specifically administered following senescence-inducing radio- or chemotherapy are still awaited for.

Better evidence than for senolytics is available for synergistic effects of potential senostatics with established tumour therapies. With respect to dietary restriction, a meta-analysis evaluated its impact across multiple cancer types and through a variety of preclinical rodent tumour models [107]. Overall, dietary restriction resulted in a 75.5% reduction in tumour incidence. Part of this beneficial effect might be due to reducing systemic chronic inflammation: increasing preclinical and human evidence suggests that dietary restriction reduces inflammation [108,109]. The reduction of energy intake reduces the amount of adipose tissue, a major endocrine organ that secretes pro-inflammatory factors including leptin, adiponectin, monocyte chemo-attractant protein-1, tumour necrosis factor, and interleukin-6 [110]. Short-term fasting also improves outcomes of chemotherapeutic treatment with etoposide [111], mitoxantrone, oxaliplatin [112], cisplatin, cyclophosphamide, and doxorubicin [113], in transgenic and transplant mouse models of neuroblastoma, fibrosarcoma, glioma [114], melanoma, and breast and ovarian cancers. Finally, alternate day fasting has been shown to enhance the radiosensitivity of mammary tumours in mice [115,116].

The dietary restriction mimetic metformin has long been prescribed for the treatment of Type 2 diabetes and polycystic ovary syndrome. In recent years, metformin has been demonstrated to function as a senostatic, inhibiting the pro-inflammatory secretory phenotype of senescent cells [73]. This appears to operate through inhibition of NF-κB signalling, with inhibition of nuclear translocation of NF-κB complexes, as well as activation of NF-κB signalling in response to lipopolysaccharide in fibroblasts and macrophages. The pro-growth effect of conditioned media from senescent cells on prostate cancer cells was reduced in media from senescent cells treated with metformin [73].

There is considerable epidemiological evidence on cancer incidence and treatment response in patients taking metformin. Diabetics taking metformin displayed reduced rates of some cancers (breast, lung and colorectal) compared to diabetic patients under other treatments, and a slight reduction in all types of cancer when compared to the general population [117]. Analysis of metformin use after lung cancer diagnosis has shown an improvement in overall survival and progression free survival [118]. However, studies and meta-analyses vary with respect to the effect size of metformin on survival benefits. They differ by cancer stage, sub-type, and patient group with stronger effects in East Asian patients possibly driving significance while Western patients show more heterogeneous results especially in lung and pancreatic cancers [[118], [119], [120], [121], [122], [123]]. A meta-analysis of metformin use in diabetics by Gandini et al. [119], focusing on confounders and biases such as body mass index, showed a reduction in overall cancer incidence and mortality with metformin use when adjusted for body mass index. With all studies considered this decrease was even larger, suggesting that the weight loss under metformin treatment regimens may also play a role [119]. However, the differences between studies and populations were considerable, suggesting possible reductions in cancer mortality at sites such as lung and liver cancers, but not others [119].

A number of studies have shown improvements in response to radiotherapy with metformin, with a reduction in three year biochemical relapse rates in localised prostate cancer [124] and in pathologic complete response rates in esophageal [125] and rectal [126] adenocarcinoma. In mice, metformin has been shown to both inhibit growth of non-small cell lung cancer cells and xenografts, and sensitising them to irradiation. Metformin treatment acted synergistically with irradiation, leading to increased DNA damage signalling, growth arrest, inhibition of mTOR signalling and increased apoptosis markers in tumours [127]. In prostate cancer xenografts treatment with metformin inhibited oxygen consumption by tumour cells and increased tumour oxygenation; in mice treated with subcurative IR doses, metformin did not have a significant effect alone, but acted synergistically with IR to slow xenograft growth [124]. Low concentrations of Metformin alone have been suggested to induce senescence in cancer hepatoma cells, while higher doses promote apoptosis [128]. In cell culture, metformin treatment following irradiation led to increased apoptosis of nasopharyngeal carcinoma [129] and hepatoma cancer cells [130].

The mechanisms by which metformin enhances radiosensitivity of various cancers are not clear. While we suggest a net senolytic activity in vivo (see Section 3 above) as potential root cause, others assumed that it may act through inhibition of double-strand break repair proteins (or an increase in initial damage generated by irradiation), leading to increased damage and apoptosis of tumour cells following irradiation. This would then suggest that metformin would increase damage, and senescence, in non-target cells. However, while metformin combined with irradiation reduced G2/M arrest, promoted sub-G1 phase cell frequencies and increased ROS levels, all indicating apoptosis in hepatocellular carcinoma cells, it only showed a moderate effect in non-cancerous hepatocytes [131]. Additionally, there is evidence for a radio-protective effect by metformin on cultured human normal blood lymphocytes [132]. In mice, metformin prior and immediately after whole body irradiation ameliorated long-term hematopoietic stem cell injury [133]. Similar effects have been seen in other cultured cells, with metformin and irradiation leading to lower levels of senescent cells and inflammatory cytokines compared to irradiation alone [134]. Metformin has also been shown to reduce lung fibrosis, immune infiltration and structural changes following high dose lung irradiation in mice [135], which may be due to a similar anti-senescence effect, as senescence in lung cells has been associated with these changes and chronic lung disease [58,136].

In conclusion, while the mechanisms of action for potential senostatic interventions are far from clear, synergisms with conventional chemo- and radiotherapy are strongly suggested by the available data. This warrants further translational research, given the generally significantly better safety profiles of senostatic as compared to senolytic interventions.

## 6 Anti-senescence to relieve frailty and multimorbidity in long-term cancer survivors

The numbers of cancer survivors is rising due to improvements in treatment outcomes and population size. Although many survivors were already in middle to advanced age at the time of diagnosis, a substantial number of children and adolescents become long-term survivors of cancer. In developed countries such as the United States and United Kingdom, the vast majority (83–84%) of children and adolescents survive for ≥ 5 years post diagnosis (although this varies considerably by cancer subtype); with survival rates remaining relatively constant (81.3%) over the past 10 years [137].

Long-term survivors of childhood and adult cancers undergo a wide-range of negative health and quality of life outcomes that lead to increased frailty and multi-morbidity compared to the general population [3,4,138]. As reviewed by Robison et al. [3], survivors who have undergone radiation therapy see both increased risk of secondary neoplasms and a wide-range of dose-dependent organ-specific effects, including cardiovascular, gastrointestinal, pulmonary, hepatic, musculoskeletal and neurological effects. Similar effects are seen with chemotherapeutic agents, especially those that directly target DNA such as alkylating agents and anthracyclines, generally increasing with cumulative dose [3]. These adverse effects can manifest soon after treatment, but also with considerable latency, and in total represent a premature ageing phenotype (see Cupit-Link et al. [4] for review); young adult survivors of childhood cancer show an 8.4 fold increase in frailty compared to siblings without cancer. Indeed, they show comparable rates of frailty to elderly people living in the community, with increased co-morbidity and a 2.76 fold increase in mortality of frail individuals [139]. Long-term follow up of adult survivors of childhood cancer showed worse general and mental health, with higher rates of functional impairment compared to siblings [140]. While long-term longitudinal data continue to be collected, it appears that cancer survivors experience increased multi-morbidity and frailty and reduced lifespan compared to the general population [3,4]. Taken together, these data suggest an acceleration of ageing in cancer survivors [4], as such, exploring the use of identified anti-ageing compounds in cancer survivors is of interest.

**Fig. 3:**
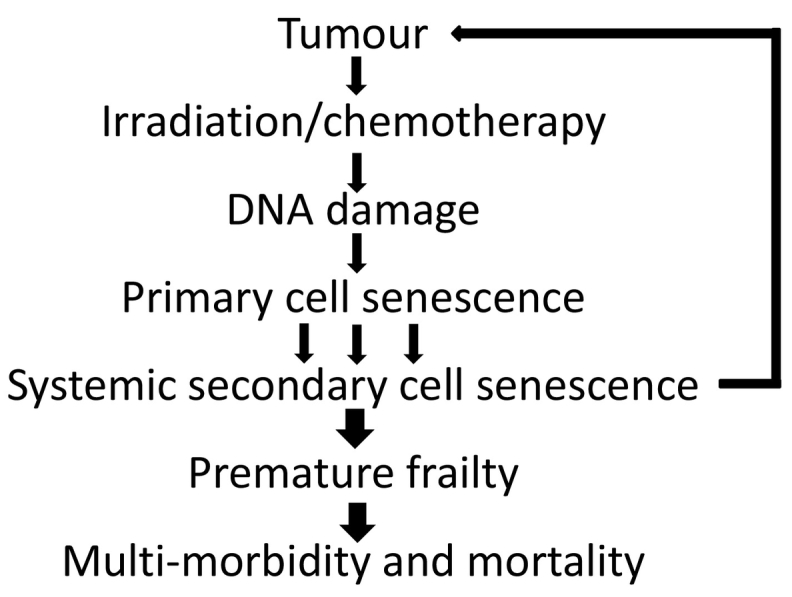
A testable hypothesis for the pathogenesis of therapy-induced frailty and multimorbidity. We propose that primary, therapy-induced senescence and its perpetuation by bystander effects is the root cause for accelerated ageing and thus for premature frailty, multi-morbidity and enhanced mortality in long-term cancer survivors. If correct, anti-senescence interventions (using senolytics or senostatics) should at least prevent, if not cure, this syndrome.

Recent reviews proposed a number of biological processes as candidate drivers of therapy-induced premature ageing, namely cell senescence, telomere attrition, epigenetic alterations, stem cell exhaustion and somatic mutations and macromolecular (DNA) damage including loss to mitochondrial DNA fidelity [4,141]. Given that most of these are part of the senescent phenotype (see Section 2 above) and given recent evidence of cell senescence as the causal factor in a wide range of (premature) ageing phenotypes (Table 1), we propose cell senescence as the focal pathogenetic mechanism of therapy-induced premature frailty and ageing (Fig. 3). We hypothesize that DNA-damaging radio- or chemotherapies induce a primary surge of senescent cells, both tumour and non-tumour cells. While these senescent cells will be ablated by the immune system within weeks to months, this time frame is sufficient to start spreading of senescence from the originally targeted cells [39], which is sufficient to cause loss of physiological resilience [46] and to perpetuate an enhancement of senescence that finally overwhelms the capacity of immunosurveillance systems. This hypothesis suggests that long-term cancer survivors will not only carry higher loads of senescent cells at steady state, but will also suffer from persistent higher rates of accumulation of such cells, presumably in relation to the severity of their premature ageing.

If this hypothesis is true, it would follow that a one-off senolytic intervention at an early time point after tumour therapy should be sufficient to reduce senescent cell frequencies to pre-treatment levels, block accelerated senescence accumulation and persistently prevent premature ageing and frailty. First data in mice show that various senolytic interventions reduce or postpone the onset of acute radiation- or chemotherapy-induced disease [56,64,65]. Preliminary data (Miwa et al. in prep) suggest that senolytics given at one month after sub-lethal whole-body irradiation are sufficient to maintain frailty for long times at levels similar to unirradiated controls and to efficiently block tumour incidence. It is however still completely unclear whether established premature frailty can be successfully treated with senolytics at late time points after therapy. However, it might be expected that late senolytic treatment should at least be able to slow further progression of accelerated ageing. Given that presently there is no treatment other than lifestyle counselling on offer for long-term cancer survivors, there is an urgent need to progress towards clinical trials. An interventional trial in hematopoietic stem cell transplant survivors (NCT02652052) is now in the recruiting phase to investigate the impact of a senolytic therapy with the combination Dasatinib and Quercetin on frailty over a 6 months follow-up period. Moreover, a phase II trial has been started to measure whether the senolytic fisetin may alleviate frailty, inflammation, and related measures in older adults (NCT03675724).

Regarding the use of senostatic agents against premature frailty, there is promising data from mice. Long-term treatment with metformin in male C57Bl/6 mice showed an extension of both healthspan and lifespan, but significant toxicity at very high doses, far in excess of reported ‘therapeutic doses’ and serum C_max_ levels in human patients [142]. Reported improvements, including changes in insulin sensitivity and cholesterol, gene transcription patterns, mitochondrial function, cognition and lower tumour incidence, were similar to those seen under caloric restriction, despite there being no reduction in calorie intake [142] [143]. Gender and strain dependent effects have been reported. For instance, a reduction in tumour rates following metformin was reported in female 129/Sv mice, but no effect on tumour incidence and in fact a reduced lifespan was seen in males [144].

In older diabetic men, metformin usage was associated with a reduction in age-related co-morbidity and frailty, with considerable effects in patients clustered as High Cancer and High Frailty risk [135]. A meta-analysis by Campbell et al. of metformin studies suggested that it may have a protective effect against all-cause mortality and age-related diseases [117]. Diabetics using metformin show a reduction in all-cause mortality compared to diabetics using other treatments; while this may be driven by metabolic alterations (as the effect is even more pronounced when compared to diabetics only taking insulin), there is still a reduction in all-cause mortality when comparing diabetics using metformin to the general and non-diabetic population not using metformin [117]. Additionally, diabetics using metformin show a reduction in a number of age-related morbidities, with reduced risk of fracture relative to rosiglitazone treatment in women and metformin users having a lower risk of fracture compared to the general population, with the effect increasing with long term treatment [145]. When compared to other diabetics, those using metformin for 6 or more years show lower levels of cognitive impairment [146]. In diabetic patients, lower rates of frailty syndrome and better performance in balance and muscle strength tests were seen in those treated with metformin [147]. Similarly, in a larger study of US veterans, metformin users showed significantly lower risk of frailty (OR 0.66, 95%CI 0.61–0.71) which was associated with lower mortality [148].

However, there are still too few data on the effect of metformin on frailty and multi-morbidity in cancer survivors, and most of the available data are limited to overall and cancer specific survival. Given the background of most studies on diabetes, multi-morbidity is often corrected for, rather than looked at specifically. Early (phase II – III) clinical studies on the effects of metformin on frailty are now coming underway (NCT02570672, NCT03451006, NCT02325245). Large clinical studies in which metformin is being tested for its capacity to reduce the onset of age-related multi-morbidity (the TAME trial, [149]) will provide better evidence as to its potential to treat cancer therapy-related frailty and premature ageing.

## 7 Outstanding questions

Combination tumour therapies with senolytics as second-line adjuvant therapeutics show significant promise. However, specificity and sensitivity of senolytics are not sufficiently well known. This is already so for senescent cells arising from different normal tissues, and will be more pertinent for senescent tumour cells harbouring multiple genetic and epigenetic aberrations, frequently in genes highly relevant for efficient apoptosis induction.

An important and so far essentially un-tackled issue for combination therapies involving senolytics will be the question of timing. Obviously, for senolytic interventions to be most effective, cells should have reached a mature senescent phenotype following first-line therapy, and tumour cells should not yet have escaped from senescence. Patients should have sufficiently recovered from first line therapies. A starting point to address this question might be that both the induction of a mature senescent phenotype following irradiation [22] and recovery from acute radiation sickness typically takes around 3 to 4 weeks.

The type(s) of tumour that should primarily be tested in combination therapies with senolytics is another important issue. We suggest diffuse high-grade glioblastoma because they are not amenable to surgery and success rates for conventional combined radio-and chemotherapy are very low and have not improved for decades. It seems very well possible that therapy-induced tumour and niche cell senescence is a significant driver of the essential limiting factor, tumour relapse.

In the context of senostatic interventions, clarification of the relevant mechanism(s) of action appears to be the most relevant issue. While it appears that DR, mTOR inhibitors or metformin reduce local and/or systemic inflammation, it is far from clear whether this is their primary mode of action or a secondary consequence of, for instance, reduction of senescence. Do they actually reduce numbers of senescent cells in a tumour therapy context as suggested in Section 3 above? How important are metabolic impacts? Is there a single primary target or are these interventions effective because they target multiple diverse mechanisms in parallel?

Finally, if anti-senescence interventions are ever to become relevant in acute tumour therapy and long-term post-tumour care, the availability of senescence markers that can be assessed in humans will be crucial. At present, this is largely an unmet need. It is unclear whether and to what extent blood-based biomarkers for immunosenescence or inflammation might be informative for the senescence status in other tissues, and non-invasive markers for cell senescence do not exist to our knowledge.

## Search strategy and selection criteria

Data for this Review were identified by searches of MEDLINE, PubMed, and references from relevant articles using the search terms “senescence”, “senolytic”, “senostatic”, “cancer”, “suvivor” and related search terms as well as by searching based on names of investigators in the field. Abstracts and reports from meetings were not included. Only articles published in English between 1980 and 2019 were included.

## Acknowledgements

Work leading to this review has been funded by CRUK Pioneer Grant C12161/A24009 to TvZ and a MRC- Arthritis Research UK Centre for Integrated Research into Musculoskeletal Ageing (CIMA) Translational Grant to SM.
